# Overcoming heparin resistance in pregnant women with antithrombin deficiency: a case report and review of the literature

**DOI:** 10.1186/s13256-018-1711-2

**Published:** 2018-06-16

**Authors:** Panagiotis Tsikouras, Anna Christoforidou, Anastasia Bothou, Dorelia Deuteraiou, Xanthoula Anthoulaki, Anna Chalkidou, Stefanos Zervoudis, Georgios Galazios

**Affiliations:** 1 Department of Obstetrics and Gynecology, Democritus University of Thrace, Alexandroupoli, Greece; 2 Department of Haematology, Democritus University of Thrace, Alexandroupoli, Greece; 3 Department of Obstetrics and Mastology, Rea Hospital, Athens, Greece

**Keywords:** Antithrombin deficiency, Pregnancy, Pregnancy loss, Heparin resistance

## Abstract

**Background:**

The risk of thromboembolic events during pregnancy in patients with antithrombin deficiency is increased. Preventing thromboembolic events during pregnancy in the case of antithrombin deficiency is still a matter of concern.

**Case presentation:**

We present a case of a 19-year-old primigravida Greek Pomak woman, who was diagnosed as having congenital antithrombin deficiency. She had a history of recurrent miscarriages and a family history of thrombosis. She was managed with adjusted doses of low molecular weight heparin throughout her pregnancy, with regular anti-Xa and antithrombin level monitoring. Prior to delivery and for 4 days after delivery she received human antithrombin III concentrate. She delivered a small for gestational age baby with no other complications. She required an increased dose of heparin due to heparin resistance.

**Conclusions:**

Antithrombin deficiency is associated with an increased risk of venous thromboembolic events with a 50% risk of thromboembolic events before the 50th year of life. It is a rare condition, so data concerning the optimal management during pregnancy are limited. The selection of patients who should receive low molecular weight heparin prophylaxis as well as dose intensity and monitoring are discussed. In our patient a conventional low molecular weight heparin dose proved to be inadequate at least at the laboratory level.

## Background

Throughout pregnancy, a state of hypercoagulability is maintained, as reflected by the increased coagulation factors (I, II, VII, VIII, IX, XII, and von Willebrand factor), reduced protein S, and inhibition of fibrinolysis. There is no significant change in plasma levels of protein C or antithrombin (AT) throughout pregnancy [1]. Pregnant patients with thrombophilia have a significantly higher risk of developing thromboembolic disease [2–4]. The thrombotic risk of a pregnant woman depends mainly on her personal and family history of thrombosis, the existence of other medical comorbidities, her body mass index (BMI), her history of thrombophilia, and other factors and should be repeatedly assessed both antenatally and postnatally [5].

Thrombophilias are congenital or acquired blood coagulation disorders that predispose to thromboembolic disease [6–8]. The term is mostly used in relation to venous thromboembolic events (VTE). In many cases, a triggering event precedes the development of VTE such as surgery, trauma, immobilization, the use of orally administered contraceptives, pregnancy, and puerperium. Among inherited thrombophilias, AT deficiency was the first to be described; AT deficiency belongs to the high risk thrombophilias, along with protein C and protein S deficiencies and homozygosity or compound heterozygosity for factor V (FV) Leiden mutation and prothrombin mutation G20210A. High risk thrombophilias are associated with an odds ratio for VTE of up to 43 in pregnant women [4].

We present a case of a 19-year-old Greek Pomak woman, primigravida, who was diagnosed with congenital AT deficiency, following a history of pregnancy loss. We focus on the challenges associated with this disorder.

## Case presentation

Our patient was a 19-year-old primigravida Greek Pomak woman who was recently diagnosed as having hereditary AT deficiency. She had been previously referred for thrombophilia testing, due to a history of two first trimester pregnancy losses. She had no history of deep vein thrombosis (VTE), but her mother had suffered from postpartum VTE at a young age. Her basic screening for thrombophilia was normal: protein C, free protein S, AT, activated protein C (APC) resistance, lupus anticoagulant, FV Leiden, factor II (FII) G20210A mutation, fasting serum homocysteine, anticardiolipin antibodies, anti-beta-2 glycoprotein 1 (anti-b2 GP1) antibodies; however, she showed an AT activity of 51% (normal range 70–120%; chromogenic Liquid Antithrombin; Instrumentation Laboratory, Milano, Italy). Her mother and two out of three of her siblings were also found to have AT deficiency, so a diagnosis of hereditary heterozygous AT deficiency was established. AT antigen testing was not available so we cannot classify the disorder as type I or II deficiency.

Three months after diagnosis she was pregnant again. We decided to manage her with adjusted dose of low molecular weight heparin (LMWH) throughout pregnancy due to the high incidence of fetomaternal complications in this disorder and our patient’s history of miscarriages. She was monitored monthly with d-dimers, AT activity, and anti-Xa measurements (liquid anti-Xa, one-stage chromogenic assay with no exogenous AT; Instrumentation Laboratory, Milano, Italy). After titrating tinzaparin dose, using chromogenic anti-Xa activity, she continued with a daily dose of 14,000 IU applied subcutaneously. With this dose the peak anti-Xa activity ranged between 0.46 and 0.79 IU/ml during the first 6 months of pregnancy, which was in great discordance with her body weight of 50 kg. This was attributed to the well-known heparin resistance phenomenon in patients with AT deficiency. During the last trimester anti-Xa activity dropped and ranged between 0.23 and 0.45 IU/ml. An attempt to raise the heparin dose did not result in significant increase in anti-Xa, but further decreased the AT levels; so we resumed the 14,000 IU dose. Throughout pregnancy d-dimers were low (93–317 μg/L) and AT was 33–35% until the 28th week, rising to 46–57% thereafter. Her pregnancy was uneventful. A cesarean section was scheduled at the 39th week due to breech presentation of the fetus. The last tinzaparin dose was given 24 hours before surgery. Prior to delivery, AT activity was 54%. In order to overcome the risk of thrombosis, 3 hours before delivery she received Kybernin P (human AT III concentrate; CSL Behring) prophylactically at a dose of 3000 IU intravenously administered, calculated according to current recommendations as follows: concentrate dose = (120% − current AT(%)) × body weight (kg) divided by 1.4. She proceeded to have general anesthesia and received tinzaparin subcutaneously 8 hours later at the conventional dose of 4500 IU. She delivered a healthy, 2610 g weight, small for gestational age male baby, who was also tested a year later and was found to have normal AT levels. There was no increased bleeding during and after caesarean section. Her AT level 2 hours after infusion was 112% and trough level the next day was 65%. Functional AT levels were measured daily prior to each dose of AT and levels were maintained between 60 and 100% by using approximately 66% of the initial AT dose or 2000 IU. We planned to administer AT for 6 days, according to various literature data, but in total she received AT for 4 days because she developed an allergic reaction after the fifth dose, so AT was discontinued and tinzaparin increased at the prior dose of 14,000 IU/day. She was discharged 6 days after delivery without complications and tinzaparin 4500 IU daily was continued for 6 weeks postpartum.

## Discussion

Pregnancy, puerperium, hormonal contraception, and hormonal replacement therapy are considered hypercoagulable states. In women with known thrombophilias these situations need expert consultation from a hemostasis specialist, especially in more severe disorders like AT deficiency, antiphospholipid syndrome, protein C or S deficiency, and the homozygous carriers of FV Leiden and FII G20210A mutations.

AT is a serine proteinase inhibitor that slowly inactivates thrombin as well as coagulation factors IXa, Xa, XIa, and XIIa in the absence of heparin, but the process is greatly accelerated in the presence of heparin. As the *in vivo* therapeutic antithrombotic effect of heparin is mediated by AT, heparin becomes ineffective in AT-deficient individuals. Inherited AT deficiency is a rare disorder, with a prevalence between 1/500 and 5000 [9, 10] in the general population and is caused by various dominant somatic mutations on the *SERPINC1* gene. It may also be acquired, for example in hepatic failure, nephrotic syndrome, metastatic tumors, or disseminated intravascular coagulation. Congenital AT deficiency is further classified as type I or quantitative deficiency and type II or functional deficiency. In type I both the antigenic and the functional protein are low, whereas in type II a dysfunctional protein is produced and only AT activity is below normal. AT deficiency is associated with a high risk of VTE with a 50% risk of thromboembolic events before the 50th year of life. Due to the rarity of the disorder there is limited literature concerning the optimal management of thrombotic events and prophylaxis during predisposing conditions and certainly no randomized trials for either anticoagulation or AT concentrates in these patients.

AT deficiency is associated with obstetric complications and serious risks for both the mother and the fetus [11]. Data interpretation of study results are complicated by the inclusion of non-controlled individuals, for example women with or without a personal or family history and the rarity of this condition [12]. The relative risk for venous thromboembolism in pregnancy was 10 and 13, respectively in two case-control studies [2, 13]. Nearly 50% of the cases occurred postpartum [14]. Despite anticoagulation prophylaxis there are cases of women who received unmonitored doses of heparin during pregnancy at either prophylactic or therapeutic schedules and nevertheless suffered a thrombosis, sometimes a fetal one [15]. This is attributed to heparin resistance and can be overcome with the concomitant administration of AT. Besides thrombosis, AT deficiency is associated with pregnancy loss, especially when no thromboprophylaxis is used [14, 16–18]. This association is not strongly supported by studies [19, 20], but results could be misleading due to the rarity of the condition in relation to other inherited thrombophilias such as FV Leiden mutation [21]. Rogenhofer *et al.* stratified pregnant women according to their personal and family history of VTE and their recommendations ranged from observation only to therapeutic anticoagulation, with AT concentrates reserved for cases where anticoagulation is withheld because of surgery or bleeding [15]. The authors commented that they had not provided recommendations on optimal AT target level or prophylactic anticoagulant dosing because it was outside the scope of the panel.

Our patient had a family history of venous thromboembolism and two first trimester pregnancy losses at a very young age. The presence of family history increases the thrombosis risk [22, 23] and according to the previous mentioned recommendations [21], LMWH should be prescribed in prophylactic or adjusted dose antepartum and/or postpartum, considering the presence of additional risk factors. Although miscarriages in adolescent pregnancy are not rare, an association with our patient’s history of AT deficiency could not be ruled out; so, we decided to manage her with “intermediate” dose of LMWH, for example to target an anti-Xa activity between 0.5 and 1 IU/ml or 50% of the full treatment dose, according to the Royal College of Obstetricians and Gynaecologists guidelines for women with AT deficiency and previous VTE [5].

This proved to be unattainable with weight-based dosing and dose escalation did not linearly increase anti-Xa, which was 0.79 IU/ml at the highest heparin dose. Anti-Xa- based dosing for LMWH is not uniformly accepted and is mostly suggested in obese pregnant women receiving full anticoagulation [12]. In our case even the therapeutic tinzaparin dose of 175 IU/kg would translate into 9000 IU per day which proved to be far from ideal for this patient.

Regarding the peripartum use of AT there are two agents available: the human plasma-derived concentrate and the recombinant AT (ATryn, not available in Greece). Recombinant AT is used as a continuous infusion and its efficacy was studied in two small single arm studies in surgical and pregnant patients with hereditary AT deficiency [24, 25]. In these studies AT was used for a mean of 3 or 7 days respectively, and no VTE was recorded. The plasma-derived factor has also been studied in small case series and it is generally used at a loading dose of (desired minus current AT(%)) × body weight (kg) divided by 1.4, followed by maintenance with 60% of the loading dose given every 24 hours, in order to maintain AT levels between 80 and 120% [26]. AT concentrate proved to be safe and effective, although the duration of its administration varied significantly [26]. AT is usually prescribed for 6 to 9 days after delivery [27]. In general, AT is recommended in pregnant women with personal history of VTE or new onset VTE, in some cases with strong family history and prior to delivery [26].

There are still many unanswered questions regarding the optimal management of pregnant women with AT deficiency, particularly the optimal dose of heparin, the role of anti-Xa measurement, the management of heparin resistance, the indications of AT concentrates, and the duration of their use [28]. In our case, we prescribed intermediate, anti-Xa monitored doses of LMWH antepartum. We also administered plasma-derived AT concentrate at the time of delivery and for 4 days postpartum (although 6 days were planned), along with 6 weeks of fixed-dose prophylactic LMWH. We are aware that our practice may be considered somehow intensive, but until further studies define the risk factors for VTE in these patients and the optimization of anticoagulation beyond cost-effectiveness, we believe that it is a reasonable and effective approach to this rare condition.

## Conclusions

In conclusion, considering the limited literature, the management of pregnant women with inherited AT deficiency should be individualized, according to the risks of each individual patient. Close monitoring with proper anti-Xa activity assays can help overcome heparin resistance. We believe the importance of this case is more clearly explained in the following: the existing literature does not define the optimal heparin dose. The use of anti-Xa monitoring, although controversial, is a useful tool in cases of heparin resistance. This is the only published case with details on how the target anti-Xa level can be achieved and details on the way AT levels change in relation to dose adjustment. We also provide a literature background on the use of various AT concentrates in pregnancy, which is really limited. More studies are needed to clarify the risk of miscarriage in these patients.
